# The Self-Assembly and Design of Polyfunctional Nanosystems

**DOI:** 10.3390/ijms22042223

**Published:** 2021-02-23

**Authors:** Ruslan Kashapov, Lucia Zakharova

**Affiliations:** 1 A.E. Arbuzov Institute of Organic and Physical Chemistry, FRC Kazan Scientific Center of RAS, 8 Arbuzov Street, Kazan 420088, Russia; 2 A.E. Arbuzov Institute of Organic and Physical Chemistry, Kazan National Research Technological University, 68 Karl Marx Street, Kazan 420015, Russia

## Abstract

The current task of the molecular sciences is to create unique nanostructured materials with a given structure and with specific physicochemical properties on the basis of the existing wide range of molecules of natural and synthetic origin. A promising and inexpensive way to obtain nanostructured materials is the spontaneous self-assembly of molecular building blocks during random collisions in real dispersive systems in solution and at interfaces. This editorial aims to summarize the major points from the 11 scientific papers that contributed to the special issue “The Self-Assembly and Design of Polyfunctional Nanosystems”, assessing the modern self-assembly potential and strategies for maintaining sustainable development of the nanoindustry.

Molecular self-assembly is a phenomenon that is important from both scientific and applied points of view. Spontaneous self-organization of molecules is a prime example of self-assembly into supramolecular structures that occurs in nature and in various useful applications. In recent years, impressive advances have been made in understanding the intricacies of self-assembly and the functioning of self-assembling systems. This special issue covers a wide range of cutting-edge research topics related to the design of functional systems based on molecular self-assembly mechanisms.

The special issue contains works that examine the self-assembly of various natural compounds. In particular, the mixed self-assembly of peptides was studied to find a complementary peptide for co-assembly with C-peptide, which is a biomarker of diabetic neuropathy [1]. Among the three nonapeptides containing tryptophan and lysine residues, the tryptophan-containing peptide interacted most efficiently with unaggregated C-peptide, with a subsequent implementation of co-assembly. This may be related to the ability of tryptophan fragments to stabilize β-hairpin peptides due to hydrophobic interactions. The approach presented in this work is of great importance for inhibiting the folding of amyloid β-42 polypeptide into toxic amyloid fibrils, which are the cause of Alzheimer’s disease.

The conformationally flexible ligand based on bispicolylamine is an excellent gelling agent in acetonitrile, in which gels with typical fibrillar morphology were formed [2]. This polymer was modified with L-alanine and dodecyl chains and formed complexes with Zn^2+^. NMR spectroscopy proved that hydrogen bonds between peptide groups and π–π stacking between aromatic fragments were the main driving forces in gel formation in the absence and presence of Zn^2+^, respectively. Moreover, in both cases, the supramolecular gels had a single-layer lamellar structure regardless of the presence of Zn^2+^, but the elasticity and viscosity of the gels decreased only in the presence of Zn^2+^. The effects identified in this paper can provide helpful information for use when designing materials to address degradability deficiencies.

The formation of fibrils may be important for the development of coatings for biomaterials in contact with bones. Fibrils coated with β-lactoglobulin from whey protein isolate, a by-product of the dairy industry, were formed in [3]. The authors of this work obtained fibrils in an acidic medium, and the yield of fibrils increased with increasing pH from 2 to 3.5; the fibrils obtained at pH 3.5 were shorter and less straight than fibrils formed at pH 2. This difference is related to the fact that at pH 2 β-lactoglobulin denatures and hydrolyzes at 90 °C into small peptides that self-assemble into amyloid aggregates, and at pH 3.5 acid hydrolysis decreases and non-hydrolyzed β-lactoglobulin forms worm-like assemblies. It is important to note that fibrils coated with a dairy product withstand autoclave sterilization and, apparently, have a positive effect on the differentiation of human bone marrow stromal cells, regardless of the method used for obtaining fibrils.

Macrocyclic compounds can be used to develop a new generation of peptidomimetics. The synthesis of peptidomimetics based on water-soluble pillar[5]arenes containing amide-ammonium-amino acid fragments was carried out, and their binding ability with a number of optically active and achiral guests containing a sulfo group was studied [4]. Modification of pillar[5]arenes by functional fragments made it possible to freeze the conformation of macrocycles to increase their binding selectivity. Synthesized macrocycles demonstrated an excellent ability to bind (1S)-(+)-10-camphorsulfonic acid and methyl orange dye, forming equimolar complexes with both guests. Moreover, the first guest can be used as a chiral matrix for non-covalent self-assembly of chiral structures based on pillar[5]arenes due to multiple electrostatic, hydrophobic, and hydrogen bonding interactions. This research may contribute to the development of new stereoselective catalysts and transmembrane amino acid channels.

Supramolecular chirality is also widely used for polymers containing azobenzene groups, as reflected in the review paper [5]. Chirality in supramolecular systems based on azopolymers is mainly achieved due to the non-covalent asymmetric arrangement of building blocks using either liquid crystalline or amorphous azopolymers. Such structures can be efficiently constructed from azopolymers in a solid film, in a dissolved state, and in a solution of assemblies by two methods: postpolymerization self-assembly and in situ polymerization-induced self-assembly. The authors of the review emphasize that the latter method is a universal approach that causes well-defined supramolecular chirality during the polymerization, and requires a detailed study of the specific mechanisms of chirality transfer and direct visualization of facile in situ supramolecular self-assembly.

The role of open-chain and macrocyclic amphiphilic compounds in the design of effective drug delivery systems has been well summarized in another review paper [6]. Since many biobarriers (lipid membrane, bacterial cell wall, mucous membrane, corneal epithelium) and biopolymers such as proteins and nucleic acids carry negatively charged fragments, much attention is paid to cationic carriers that provide a high affinity of encapsulated drugs to target cells. In addition, the authors of this review paper highlight aspects of the clinical implementation of innovative drug delivery systems. In this context, supramolecular modification of drug nanocarriers is a promising approach to enhance drug therapeutic efficacy.

Three dimensional macrocyclic structures can be used to improve the stability of biologically active substances that oxidize easily when exposed to air, light, and/or heat. The study presented in [7] was aimed at studying the protective ability of γ-cyclodextrin in relation to ascorbic acid by the formation of ternary complexes with polyvinyl alcohol. These complexes were formed as a result of interactions between the lactone ring of an antioxidant, the hydroxyl group of a polymer, and the hydrophobic cavity of a macrocycle. The inclusion of ascorbic acid reduced the mixed self-assembly of the macrocycle with the polymer, but at the same time favorably increased the stability of the ternary system. It was found that in binary systems with ascorbic acid, γ-cyclodextrin retains its antioxidant activity, while polyvinyl alcohol exhibits the opposite effect. In ternary systems, a similar trend was observed in the change in the antioxidant activity of ascorbic acid, depending on the ratio of γ-cyclodextrin and polyvinyl alcohol. The revealed phenomena can be useful for the formation of nanoscale compositions of labile active substances.

The driving force of mixed co-assembly may be the electrostatic interaction between oppositely charged substances. Pairs of cationic and anionic surfactants are capable of spontaneously assembling into hollow vesicular structures. The thermal transition of such mixed systems with a small excess of anionic surfactant from a multilamellar structure to a single-layer structure was studied in [8]. Interestingly, the anionic surfactant SDS in a mixture with a cationic surfactant underwent dissociation as a result of an increase in temperature, which led to this transition. A cationic derivative of curdlan synthesized in another work [9] was mixed with the anionic derivative of hydroxypropyl cellulose to obtain nanoparticles of coacervate, which were formed as a result of a spontaneous and immediate self-assembly in water. The resulting nanoparticles can transport and release a hydrophobic drug, piroxicam, into mouse embryonic fibroblast cells NIH3T3 in response to temperature changes. The lower critical temperature for dissolution of nanoparticles was 41 °C, which corresponds to biomedical applications. Moreover, these nanoparticles lacked cytotoxicity, which increased by 20% upon uptake of piroxicam with an encapsulation efficiency of 94.80% and a loading efficiency of 0.73%. However, the resulting encapsulated form of piroxicam was significantly less cytotoxic than was the non-encapsulated form of this drug.

Another means of improving therapeutic activity is the creation of prodrug forms. Conjugation of paclitaxel with α-tocopherol (vitamin E) results in a hydrophobic prodrug, the anticancer efficacy of which was further enhanced by its encapsulation in nanoparticles formed using Flash NanoPrecipitation of tannic acid and iron, forming the nanoparticle core [10]. The growth of this core was stabilized by adsorption of polystyrene-b-polyethylene glycol on the core surface. The cytotoxic effect on the ovarian cancer cell line OVCA-432 was further enhanced by co-encapsulation of the prodrug with lapatinib. Given the higher drug release at pH 7 compared to that at pH 4, these nanoparticles can serve as oral dosage forms.

Synergistic therapeutic benefit is also achieved by combining docetaxel and curcumin. The work published in [11] is aimed at determining the optimal formulation of aerosol nanoemulsions containing these two biologically active components. The study involves a D-optimal mixture experimental design model, which takes into account the effects of interaction, allowing for a reduction in the time and cost of sample preparation. The actual values of the optimized blend formulations of palm kernel oil ester, safflower seed oils, lecithin, Tween 85, and Span 85 were in line with the predicted values from the modeling technique, and they showed favorable physicochemical (particle size, polydispersity index, zeta potential, pH, viscosity, osmolality) and aerodynamic (aerodynamic diameter, high percentage of fine particles in the lower airways of the lungs) properties required for inhalation therapy. Thus, the optimized formulations can potentially be used as a drug delivery system for the treatment of lung cancer.

In summary, these 11 papers, published in the special issue "The Self-Assembly and Design of Polyfunctional Nanosystems" by contributors from 16 countries, confirm the important function of self-assembly, which is of great interest to the world’s scientific community. As we can see from these works, self-assembly involves a wide range of both natural and synthetic molecules and finds many useful applications. The self-assembly of molecular building blocks meets the criteria for green chemistry as it can be classified as an alternative to organic synthesis, which can consume organic solvents, time, and energy. Today, the principles of spontaneous organization of molecules make it possible to obtain functional nanomaterials with all possible structures and properties. All this can provide the broadest opportunities for the creation and rapid commercialization of self-assembling molecular structures with different functionality.
